# Effects of paramedical counselling on anxio-depressive symptoms, perceived stress and self-esteem in male factor infertility

**DOI:** 10.1192/j.eurpsy.2022.726

**Published:** 2022-09-01

**Authors:** A. Szatmári, K. Helembai, J. Zádori, I. Kovács

**Affiliations:** 1 Faculty of Health Sciences and Social Studies, University of Szeged, Department Of Health Sciences, Szeged, Hungary; 2 Center for Assisted Reproduction, Albert Szent-györgyi Clinical Center, Faculty Of Medicine, University Of Szeged, Szeged, Hungary; 3 Szent-Györgyi Albert Medical School, University of Szeged, Department Of Psychiatry, Szeged, Hungary

**Keywords:** male infertility, anxio-depressive symptoms, paramedical counselling

## Abstract

**Introduction:**

Elevated levels of anxio-depressive symptoms and perceived stress are widely researched in case of female factor infertility; however, there is scant information on their emergence in case of male factor infertility.

**Objectives:**

The aim of the present study is to assess whether a 5-course paramedical counselling accompanying infertility treatment would have a decreasing impact on anxio-depressive symptom severity and perceived stress and would increase the level of self-esteem in infertile men.

**Methods:**

108 patients were divided into control (n = 51) and experimental (n = 57) groups, where the latter participated in the aforementioned paramedical counselling. Anxio-depressive symptom severity was measured with the Beck Depression Inventory and the Spielberger’s State Anxiety Inventory; perceived stress was registered with the Perceived Stress Scale and Brief Stress and Coping Inventory, while self-esteem was evaluated by the Rosenberg Self-Esteem Scale.

**Results:**

Participation in an infertility programme itself affected positively patients’ self-esteem and decreased their levels of depressive symptom severity (t(50) = 2.738, p = 0.009, 95%CI = 0.167 – 1.088), but an additional 5-session paramedical counselling resulted in a significant lowering of state anxiety symptoms (t(106) = -2.093, p = 0.039, 95%CI = -6.372 – 0.173) contrasted with infertile men not receiving this additional counselling.

**Conclusions:**

Conclusion: Screening for psychological factors is advisable in the course of an infertility treatment, and the implementation of an accompanying paramedical counselling focusing on the alleviation of concomitant psychopathological symptoms would be advisable among male infertile patients.

**Disclosure:**

No significant relationships.

